# Effect of the asthma–chronic obstructive pulmonary disease syndrome on the stroke, Parkinson's disease, and dementia: a national cohort study

**DOI:** 10.18632/oncotarget.23811

**Published:** 2017-12-26

**Authors:** Jun-Jun Yeh, Yu-Feng Wei, Cheng-Li Lin, Wu-Huei Hsu

**Affiliations:** ^1^ Ditmanson Medical Foundation Chia-Yi Christian Hospital, Chiayi, Taiwan; ^2^ Chia Nan University of Pharmacy and Science, Tainan, Taiwan; ^3^ Meiho University, Pingtung, Taiwan; ^4^ Pingtung Christian Hospital, Pingtung, Taiwan; ^5^ Department of Internal Medicine, E-Da Hospital, I-Shou University, Kaohsiung, Taiwan; ^6^ Management Office for Health Data, China Medical University Hospital, Taichung, Taiwan; ^7^ College of Medicine, China Medical University, Taichung, Taiwan; ^8^ Graduate Institute of Clinical Medical Science and School of Medicine, College of Medicine, China Medical University, Taichung, Taiwan; ^9^ Division of Pulmonary and Critical Care Medicine, Department of Internal Medicine, China Medical University Hospital, Taichung, Taiwan

**Keywords:** asthma–chronic obstructive pulmonary disease syndrome (ACOS), stroke, Parkinson's disease (PD), dementia, inhaler steroids

## Abstract

**Background:**

To evaluate the association of asthma–chronic obstructive pulmonary disease syndrome (ACOS) with neurodegenerative diseases (stroke, Parkinson's disease and dementia) and the role of the steroids in the neurodegenerative diseases among the ACOS cohort.

**Materials and Methods:**

Comparison of the ACOS cohort (*N* = 10,260) with the non-ACOS cohort (*n =* 20,513) based on the patients aged ≧40 years in the National Health Insurance Research Database from January 1, 2000 to December 31, 2010. These patients follow up to diagnosis of neurodegenerative diseases or the December 31, 2011; using multivariable Cox proportional hazards models.

**Results:**

After adjustment for potential confounders, the [adjusted hazard ratio (aHR), 95% confidence interval (CI)] in the ACOS cohort were [1.39, 1.28–1.50] [1.56, 1.34–1.81] and [1.43, 1.29–1.59] for stroke, Parkinson's disease, dementia; respectively. The [aHR, 95% CI] for ACOS cohort with (inhaler corticosteroids ≧0.13 gram/ oral steroids ≧0.08gram) were with less risk (all aHR<1, *p* values <0.05) for these 3 neurodegenerative diseases except Parkinson's disease with inhaler corticosteroids >0.43 gram. The risk of stroke and dementia were the lower in patients with < 250 μg/d of a fluticasone equivalent inhaler corticosteroids (aHR = 0.53, 95% CI = 0.35–0.79; aHR = 0.53, 95% CI = 0.31–0.90, respectively).

**Conclusions:**

The ACOS cohort had a higher risk of the neurodegenerative diseases. The lower dose of the inhaler corticosteroids with cumulative dose ≧0.13 gram have the less risk of stroke and dementia.

## INTRODUCTION

The increasing prevalence of frailty [1] is a concern in both developed and developing countries [2]. Frailty is associated with neurological disorders, such as stroke, dementia, and Parkinson's disease (PD) [3]. Stroke and dementia share many risk factors, including smoking-related diseases [e.g., diabetes, hypertension, hyperlipidemia, coronary artery disease (CAD), sleep disorder, cancer, tuberculosis (TB)] and alcohol-related illness. All major dementias have a vascular component, and stroke increases the risk of dementia. Further interactions may relate the diseases, including the effect of blood pressure on amyloid development and stroke increased the risk of the amyloid inflammation [4]. Similarly, the amyloidosis deposit in the brain associated with the PD [5]. Therefore, these neurological inflammation diseases (PD, stroke, dementia) may concurrently occur in the same patients.

Neurodegenerative diseases, including PD, dementia with stroke [6]. PD may be caused by either genetic or environmental factors [7], and the mechanisms may include oxidative stress, or system inflammation. Oxidative stress is presumed to be the main mechanism of neurodegeneration [8]. The hypoxemia status causes the systemic inflammation, associated [9] with Interferon gamma (IFNγ), the tumor necrosis factor-alpha (TNF-α) [10], and oxidative stress [11] leading to direct neuronal damage, as well as depletion of neurotransmitters because of the dysfunction of oxygen-dependent enzymes in chronic obstructive pulmonary disease (COPD) [12]. ACOS [13] is the concurrent occurrence of asthma [9] and COPD [14]. The high frequency of ACOS [15] with exacerbation aggravate systemic inflammation [12] and hypoxemia [16]. Therefore, the patients with ACOS may be associated with the neurodegenerative diseases [9, 16, 17], especially these patients of asthma or COPD with exacerbation [18] and deterioration of hypoxemia [17–19]. This speculation didn’t examined in detail up to today in English literature.

The inhaler corticosteroid (ICSs)/oral steroids (OSs) have the effect of the anti-inflammation and may attenuate the hypoxemia in ACOS [15]. But the relationship between the ICSs [20]/ OSs and the neurodegenerative diseases among the ACOS cohort [21] comparison with the non-ACOS cohort based on the general population were not found in previous study. Therefore, we examined the occurrence of neurodegenerative diseases [22] in the ACOS cohort, and the role of the ICSs/OSs [23] in the neurodegenerative diseases among the ACOS cohort comparison with the non-ACOS cohort. We tested these hypotheses in the general population.

## RESULTS

We included 10,260 and 20,513 patients in the ACOS and non-ACOS cohorts, respectively, with similar sex and age distributions (Table 1). In both cohorts, 57.0% of the patients were men and more than 55.1% of the patients were aged ≥ 65 years. The mean age of patients in the ACOS and non-ACOS cohorts was 65.6 (SD = 11.8) and 65.5 (SD = 11.9) years, respectively. Compared with the non-ACOS cohort, the ACOS cohort showed a higher prevalence of smoking-related comorbidities (e,g. diabetes, hypertension, hyperlipidemia, CAD, alcohol-related diseases, anxiety, sleep disorder, pneumonia and TB) and atopic related diseases, except for cancer.

**Table 1:** Comparison of demographics and history of comorbidity between ACOS and non-ACOS cohorts

	ACOS				
	No (*N* = 20513)		Yes (*N* = 10260)		
Variable	*n*	%	*n*	%	*p*-value
Sex					0.99
Female	8826	43.0	4414	43.0	
Male	11687	57.0	5846	57.0	
Age, year					0.99
40–49	2470	12.0	1235	12.0	
50–64	6736	32.8	3368	32.8	
≥65	11307	55.1	5657	55.1	
Mean (SD)^#^	65.5	11.9	65.6	11.8	0.38
Comorbidity					
Diabetes	4019	19.6	2425	23.6	< .0001
Hypertension	9659	47.1	6254	61.0	< .0001
Hyperlipidemia	5065	24.7	3271	31.9	< .0001
Coronary artery disease	5044	24.6	4065	39.6	< .0001
Alcohol-related illness	469	2.29	494	4.81	< .0001
Sleep disorder	3659	17.8	3063	29.9	< .0001
Anxiety	1371	6.68	1223	11.9	< .0001
Atopic related diseases	5586	27.2	4739	46.2	< .0001
Cancer	756	3.69	396	3.86	0.45
Tuberculosis	466	2.27	782	7.62	< .0001
Pneumonia	1005	4.90	1785	17.4	< 0.001
Medicine					
Inhaled corticosteroids (ICSs)	652	3.18	2722	26.5	< .0001
Oral steroids(OSs)	8243	40.2	7941	77.4	< .0001

ACOS, asthma–COPD overlap syndrome; Chi-square test; ^#^Student’s *t*-test.

**Figure 1:**
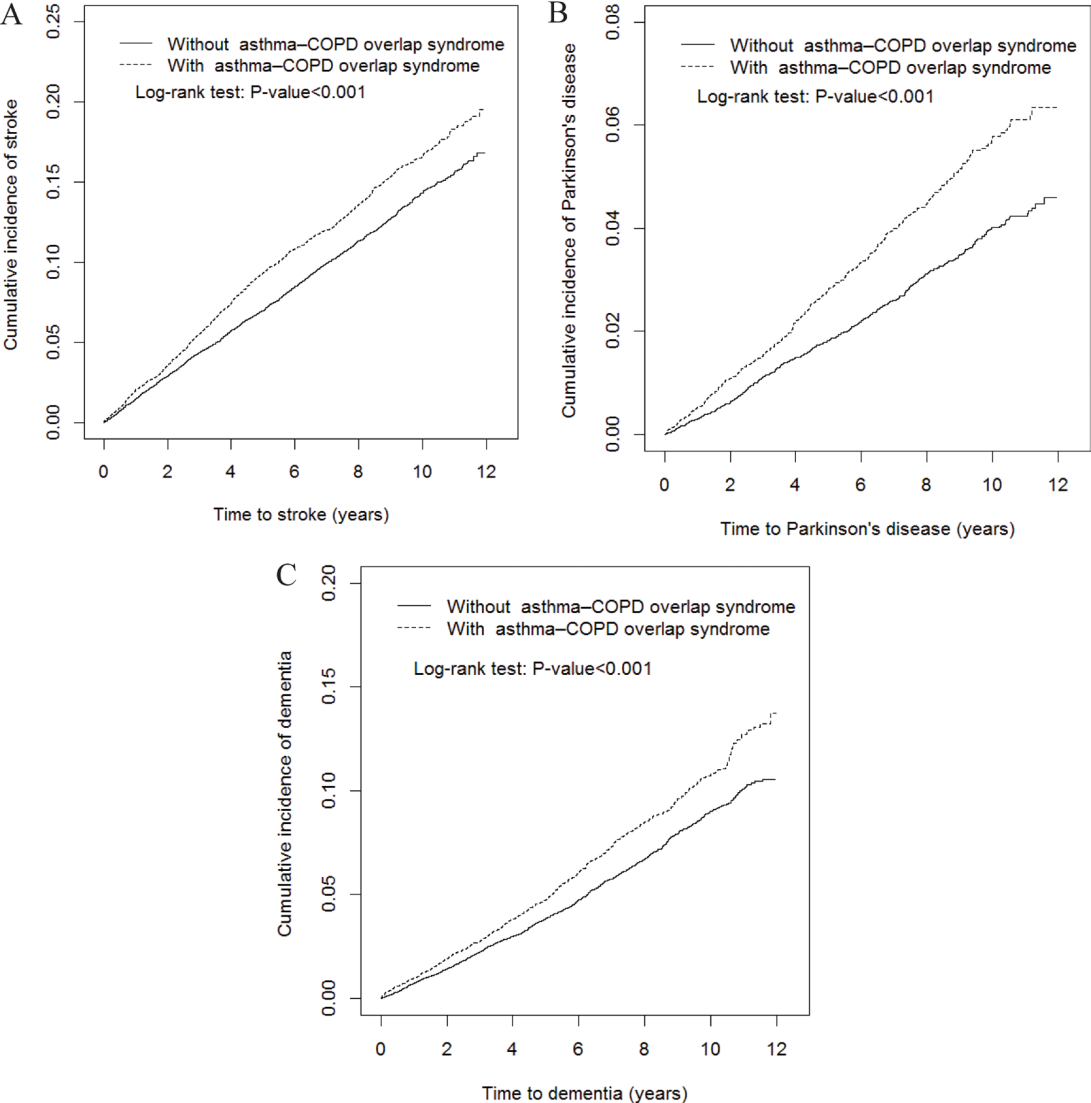
The cumulative incidence of stroke (**A**), Parkinson’s disease (**B**), dementia (**C**) in asthma–COPD overlap syndrome (ACOS) (dashed line) and non-ACOS cohorts (solid line).

Overall, the ACOS cohort had higher incidence density rates of stroke (18.5 vs. 15.1 per 1000 person-years), PD (5.72 vs. 3.87 per 1000 person-years), and dementia (11.1 vs. 8.81 per 1000 person-years) than did the non-ACOS cohort, with a crude hazard ratio (cHR) of 1.23 (95% CI = 1.15–1.32), 1.48 (95% CI = 1.30–1.69), and 1.26 (95% CI = 1.15–1.38), respectively (Table 2). The Multivariable Cox proportional hazard regression analysis revealed a significantly higher risk of stroke in the ACOS cohort [adjusted HR (aHR) = 1.39, 95% CI = 1.28–1.50] than in the non-ACOS cohort. Similar results were obtained for PD and dementia because the ACOS cohort showed a 1.56-fold (95% CI = 1.34–1.81) and 1.43-fold (95% CI = 1.29–1.59) higher risk of PD and dementia. After sex stratification, the relative risk of stroke in the ACOS cohort compared with the non-ACOS cohort was significantly higher in both women (aHR = 1.38, 95% CI = 1.21–1.58) and men (aHR = 1.38, 95% CI = 1.24–1.53). The age-specific relative risk of stroke in both cohorts was higher in patients aged 40–65 (aHR = 1.34, 95% CI = 1.11–1.60) and ≥ 65 years (aHR = 1.37, 95% CI = 1.25–1.50). The risk of stroke was significantly increased in the ACOS cohort than in the non-ACOS cohort after the stratification of patients without any comorbidities (aHR = 1.64, 95% CI = 1.21–2.22) and of those with at least one comorbidity (aHR = 1.42, 95% CI = 1.31–1.54). The risks of [PD and dementia], in ACOS cohorts aged > 65 years [(aHR = 1.56, 95% CI = 1.33–1.83); (aHR = 1.42, 95% CI = 1.27–1.58)] or with comorbidity [(aHR = 1.64, 95% CI = 1.42–1.91); (aHR = 1.54, 95% CI = 1.38–1.71)] were higher than the non-ACOS cohorts. Figures 1A–1C show the significantly higher cumulative incidence of stroke (*P* < 0.001; Figure 1A), PD (*P* < 0.001; Figure 1B), and dementia (*P* < 0.001; Figure 1C) in the ACOS cohort than in the non-ACOS cohort. In the interaction analysis, the patients without ICSs/OSs were at a higher risk of stroke, PD, dementia compared to patients with ICSs/OSs (the *P*-value of interaction < 0.001).

**Table 2:** Incidence rate and adjusted hazard ratio of stroke, Parkinson’s disease, dementia between ACOS and non-ACOS cohorts stratified by sex, age, comorbidity (no/yes), and drug used (no/yes)

	ACOS							
		No			Yes		Compared to non-ACOS cohort	
Variables	Event	PY	Rate	Event	PY	Rate	Crude HR (95% CI)	Adjusted HR† (95% CI)
Stroke								
Overall	2008	133159	15.1	1187	64030	18.5	1.23 (1.15, 1.32)^***^	1.39 (1.28, 1.50)^***^
Sex								
Female	710	59618	11.9	456	28943	15.8	1.32 (1.18, 1.49)^***^	1.38 (1.21, 1.58)^***^
Male	1298	73541	17.7	731	35088	20.8	1.18 (1.08, 1.29)^***^	1.38 (1.24, 1.53)^***^
*p* for interaction								0.13
Age, year								
40–65	394	65268	6.04	276	32069	8.61	1.43 (1.23, 1.67)^***^	1.34 (1.11, 1.60)^**^
≥ 65	1614	67891	23.8	911	31961	28.5	1.20 (1.11, 1.30)^***^	1.37 (1.25, 1.50)^***^
*p* for interaction								0.16
Comorbidity								
No	338	44025	7.68	61	6797	8.97	1.16 (0.88, 1.52)	1.64 (1.21, 2.22)^**^
Yes	1670	89133	18.7	1126	57233	19.7	1.05 (0.97, 1.13)	1.42 (1.31, 1.54)^***^
*p* for interaction								0.46
Drug used								
No	1240	78540	15.8	302	11675	25.9	1.63 (1.43, 1.85)^***^	1.50 (1.30, 1.71)^***^
Yes	768	54619	14.1	885	52355	16.9	1.20 (1.09, 1.32)^***^	1.17 (1.06, 1.29)^**^
*p* for interaction								< 0.001
Parkinson’s disease								
Overall	532	137308	3.87	380	66438	5.72	1.48 (1.30, 1.69)^***^	1.56 (1.34, 1.81)^***^
Sex								
Female	200	61102	3.27	146	30030	4.86	1.49 (1.20, 1.84)^***^	1.47 (1.15, 1.88)^**^
Male	332	76206	4.36	234	36408	6.43	1.48 (1.25, 1.75)^***^	1.59 (1.32, 1.93)^***^
*p* for interaction								0.97
Age, year								
40–65	74	66353	1.12	58	32910	1.76	1.58 (1.12, 2.23)^**^	1.45 (0.96, 2.17)
≥ 65	458	70954	6.45	322	33528	9.60	1.49 (1.29, 1.72)^***^	1.56 (1.33, 1.83)^***^
*p* for interaction								0.70
Comorbidity								
No	64	44702	1.43	11	6969	1.58	1.10 (0.58, 2.08)	1.74 (0.85, 3.57)
Yes	468	92605	5.05	369	59469	6.20	1.23 (1.07, 1.41)^**^	1.64 (1.42, 1.91)^***^
*p* for interaction								0.74
Drug used								
No	328	81341	4.03	110	12325	8.92	2.21 (1.78, 2.74)^***^	1.85 (1.47, 2.32)^***^
Yes	204	55967	3.64	270	54113	4.99	1.37 (1.14, 1.65)^***^	1.30 (1.08, 1.57)^**^
*p* for interaction								< 0.001
Dementia								
Overall	1194	135558	8.81	726	65634	11.1	1.26 (1.15, 1.38)^***^	1.43 (1.29, 1.59)^***^
Sex								
Female	487	60223	8.09	298	29604	10.1	1.25 (1.08, 1.44)^**^	1.40 (1.19, 1.65)^***^
Male	707	75335	9.38	428	36031	11.9	1.27 (1.13, 1.43)^***^	1.44 (1.26, 1.65)^***^
*p* for interaction								0.84
Age, year								
40–65	100	66337	1.51	79	32875	2.40	1.60 (1.19, 2.15)^**^	1.26 (0.89, 1.80)
≥65	1094	69221	15.8	647	32759	19.8	1.26 (1.14, 1.39)^***^	1.42 (1.27, 1.58)^***^
*p* for interaction								0.09
Comorbidity								
No	194	44392	4.37	26	6908	3.76	0.85 (0.56, 1.28)	1.38 (0.88, 2.17)
Yes	1000	91166	11.0	700	58727	11.9	1.09 (0.99, 1.20)	1.54 (1.37, 1.71)^***^
*p* for interaction								0.27
Drug used								
No	749	80234	9.34	204	12085	16.9	1.82 (1.56, 2.12)^***^	1.61 (1.37, 1.90)^***^
Yes	445	55324	8.04	522	53549	9.75	1.22 (1.07, 1.38)^**^	1.18 (1.04, 1.35)^*^
*p* for interaction								< 0.001

ACOS, asthma–COPD overlap syndrome; Drug used, including subjects with inhaled corticosteroids (ICSs) or oral steroids(OSs); PY, person-year; Rate, incidence rate (per 1,000 person-years); IRR, incidence rate ratio; Adjusted HR^†^: multiple cox model analysis including age, sex, each comorbidity, inhaled corticosteroid (ICSs), and oral steroids(OSs); ^*^p < 0.05, ^**^p < 0.01, ^***^p < 0.001.

Table 3 presents the risks of stroke, PD, and dementia in patients with ACOS who used ICSs/OSs. The aHR (95% CI) in patients with ACOS who received ICSs < 0.12 gram were 1.08 (0.89–1.32); 1.81 (1.33–2.46); 1.05 (0.80–1.36) for stroke, PD and dementia. The aHR (95% CI) of the OSs < 0.07 gram were 2.05 (1.87–2.26); 2.31 (1.96–2.73); 1.97 (1.75–2.23) for the stroke, PD and dementia. The risk of the neurodegenerative diseases were high with the OSs < 0.07 gram and PD was higher with the ICSs < 0.012 gram (the non-ACOS cohort as reference 1).

**Table 3:** The association between ACOS and drug day (per year) and average dose (per year) of ICSs, OSs, and antibiotic therapy for the risk of stroke, Parkinson’s disease, dementia

Variables	N	Event	Rate	Crude HR (95% CI)	Adjusted HR (95% CI)^†^
Stroke					
Non-ACOS cohort	20513	2008	15.1	1.00	1.00
ACOS cohort					
Without ICSs	7538	984	21.7	1.44 (1.34, 1.56)^***^	1.43 (1.31, 1.55)^***^
With ICSs (gram), per year					
≤ 0.12	910	105	14.4	0.95 (0.78,1 .16)	1.08 (0.89, 1.32)
0.13–0.43	869	52	8.60	0.57 (0.43, 0.75)^***^	0.64 (0.48, 0.85)^**^
> 0.43	1853	46	8.55	0.57 (0.42, 0.76)^***^	0.57 (0.43, 0.77)^***^
Non-ACOS cohort	20513	2008	15.1	1.00	1.00
ACOS cohort					
Without OSs	2319	326	24.3	1.61 (1.43, 1.81)^***^	1.64 (1.46, 1.85)^***^
With OSs (gram), per year					
≤0.07	2573	619	33.0	2.19 (2.00, 2.40)^***^	2.05 (1.87, 2.26)^***^
0.08–0.26	2650	109	5.97	0.40 (0.33, 0.48)^***^	0.42 (0.34, 0.50)^***^
> 0.26	2718	133	9.80	0.65 (0.55, 0.77)^***^	0.63 (0.52, 0.75)^***^
Non-ACOS cohort	20513	2008	15.1	1.00	1.00
ACOS cohort					
Without ICSs	7538	984	21.7	1.44 (1.34, 1.56)^***^	1.42 (1.30, 1.54)^***^
ICSs (fluticasone equivalent)					
Low dose (< 250 ug/d)	451	24	6.65	0.44 (0.30, 0.66)^***^	0.53 (0.35, 0.79)^**^
Middle dose (250–500 ug/d)	375	25	9.72	0.64 (0.43, 0.96)^*^	0.70 (0.47, 1.04)
High dose (>500 ug/d)	1896	154	12.3	0.82 (0.69, 0.96)^*^	0.86 (0.73,1 .02)
Parkinson’s disease					
Non-ACOS cohort	20513	532	3.87	1.00	1.00
ACOS cohort					
Without ICSs	7538	309	6.52	1.69 (1.47, 1.94)^***^	1.59 (1.37, 1.85)^***^
With ICSs (gram), per year					
≤ 0.12	910	47	6.28	1.61 (1.19, 2.17)^**^	1.81 (1.33, 2.46)^***^
0.13–0.43	869	11	1.80	0.46 (0.26, 0.84)^*^	0.53 (0.29, 0.97)^*^
> 0.43	1853	13	2.40	0.62 (0.36, 1.08)	0.65 (0.37, 1.13)
Non-ACOS cohort	20513	532	3.87	1.00	1.00
ACOS cohort					
Without OSs	20513	532	3.87	2.12 (1.74, 2.60)^***^	1.98 (1.61, 2.43)^***^
With OSs (gram), per year					
≤ 0.07	2573	218	10.8	2.77 (2.36, 3.24)^***^	2.31 (1.96, 2.73)^***^
0.08–0.26	2650	23	1.25	0.32 (0.21, 0.49)^***^	0.30 (0.20, 0.47)^***^
> 0.26	2718	23	1.68	0.44 (0.29, 0.67)^***^	0.35 (0.23, 0.55)^***^
Non-ACOS cohort	20513	532	3.87	1.00	1.00
ACOS cohort					
Without ICSs	7538	309	6.52	1.69 (1.47, 1.94)^***^	1.59 (1.36, 1.85)^***^
ICSs (fluticasone equivalent)					
Low dose (< 250 ug/d)	451	10	2.75	0.71 (0.38, 1.32)	0.83 (0.44, 1.55)
Middle dose (250–500 ug/d)	375	11	4.22	1.09 (0.60, 1.98)	1.21 (0.66, 2.20)
High dose (>500 ug/d)	1896	50	3.91	1.01 (0.76, 1.35)	1.09 (0.81, 1.48)
Dementia					
Non-ACOS cohort	20513	1194	8.81	1.00	1.00
ACOS cohort					
Without ICSs	7538	619	13.3	1.52 (1.38, 1.67)^***^	1.49 (1.34, 1.66)^***^
With ICSs (gram), per year					
≤ 0.12	910	59	7.93	0.88 (0.68, 1.15)	1.05 (0.80, 1.36)
0.13–0.43	869	25	4.10	0.47 (0.31, 0.69)^***^	0.58 (0.39, 0.87)^**^
> 0.43	1853	23	4.25	0.49 (0.32, 0.74)^***^	0.55 (0.36, 0.83)^**^
Non-ACOS cohort	20513	1194	8.81	1.00	1.00
ACOS cohort					
Without OSs	2319	213	15.3	1.76 (1.52, 2.04)^***^	1.78 (1.54, 2.07)^***^
With OSs (gram), per year					
≤ 0.07	2573	383	19.4	2.17 (1.93, 2.43)^***^	1.97 (1.75, 2.23)^***^
0.08–0.26	2650	67	3.65	0.42 (0.33, 0.53)^***^	0.44 (0.34, 0.56)^***^
> 0.26	2718	63	4.62	0.54 (0.42, 0.69)^***^	0.51 (0.39, 0.66)^***^
Non-ACOS cohort	20513	1194	8.81	1.00	1.00
ACOS cohort					
Without ICSs	7538	619	13.3	1.52 (1.38, 1.67)^***^	1.48 (1.33, 1.65)^***^
ICSs (fluticasone equivalent)					
Low dose (< 250 ug/d)	451	14	3.86	0.43 (0.25, 0.73)^**^	0.53 (0.31, 0.90)^*^
Middle dose (250–500 ug/d)	375	11	4.20	0.48 (0.26, 0.86)^*^	0.55 (0.31, 1.01)
High dose (>500 ug/d)	1896	82	6.45	0.73 (0.59, 0.92)^**^	0.85 (0.67, 1.07)

ACOS, asthma–COPD overlap syndrome; the average use day on median use is partitioned in to 2 segments by median; the average dose on median use is partitioned in to 3 segments by tertile; Rate, incidence rate (per 1,000 person-years); IRR, incidence rate ratio; Adjusted HR^†^: multiple cox model analysis including age, sex, each comorbidity, inhaled corticosteroid (ICSs), oral steroid(OSs); ^*^p < 0.05, ^**^p < 0.01, ^***^p < 0.001.

The ICSs with 0.13–0.43 gram, the aHR (95% CI) were 0.64 (0.48–0.85); 0.53 (0.29–0.97); 0.58 (0.39–0.87) for the stroke, PD and dementia. The OSs with 0.08–0.26 gram, the aHR (95% CI) were 0.42 (0.34–0.50), 0.30 (0.20–0.47), 0.44 (0.34–0.56) for the stroke, PD and dementia. The ICSs with > 0.43 gram, the aHR (95% CI) were 0.57 (0.43–0.77), 0.65 (0.37–1.13), 0.55 (0.36–0.83) for the stroke, PD and dementia. The OSs with > 0.26 gram, the aHR (95% CI) were 0.63 (0.52–0.75), 0.35 (0.23–0.55), 0.51 (0.39–0.66) for the stroke, PD and dementia. The risk of the degenerative diseases were less in the ACOS cohort except ICSs > 0.43 gram for PD (the non-ACOS cohort as reference 1).

Without [ICSs; OSs], the aHR (95% CI) were [1.43 (1.31–1.55), 1.59 (1.37–1.85)], [1.49 (1.34–1.66); 1.64 (1.46–1.85)], [1.98 (1.61–2.43), 1.78 (1.54–2.07)] for the stroke, PD, and dementia. The risk of the neurodegenerative diseases were higher in the ACOS cohort without OSs /ICSs (the non-ACOS cohort as reference 1).

Compared with the non-ACOS cohort, the risk of stroke and dementia were the lower in patients with low-dose use less than 250 μg/d of a fluticasone equivalent ICSs (aHR = 0.53, 95% CI = 0.35–0.79; aHR = 0.53, 95% CI=0.31–0.90, respectively). The ICSs with [250 μg/d-500 μg/d, > 500 μg/d]of a fluticasone equivalent ICSs, the aHR (95% CI) were [0.70 (0.47–1.04), 0.86 (0.73–1.02)], [1.21 (0.66–2.20); 1.09 (0.81–1.48)], [0.55 (0.31–1.01), 0.85 (0.67–1.07)] for the stroke, PD, and dementia.

## DISCUSSION

The ACOS cohort showed a higher risk of neurodegenerative diseases, namely stroke, PD and dementia regardless of age, sex, and comorbidities. These diseases were associated with cerebrovascular diseases [24]. Therefore, the risk of stroke was higher in patients with ACOS, even without comorbidities or when patients were young adults (≧ 40 years < 65 years). Kumbhare et al. [25] reported stroke associated with relatively young adults of ACOS cohort in real world, consistent with our findings. Meanwhile, the ACOS cohort without ICSs/OSs use have the higher risk of the neurodgenerative disaeases. Furthermore, the ACOS cohort with ICSs > 0.13 gram or OSs > 0.08 gram have the less risk of the neurodegenerative diseases. These findings imply that the ACOS associated with the risk of the neurodegenerative diseases; the ACOS with higher level of ICSs/OSs may have the less incidence of the neurodegenerative diseases.

Comorbidities (e.g. TB) [26] or alcohol consumption [27] were associated with the risk of PD. Meanwhile, atherosclerosis-related diseases (e.g. diabetes, hypertension, hyperlipidemia, CAD) and hypoxemia-related diseases (e.g. pneumonia, sleep disorder) play a role for neurodegenerative diseases. The additive effect of the [29] alcohol-related diseases, atherosclerosis-related diseases and hypoxemia-related diseases may have associated with the risk of neurodegenerative diseases. The higher risk of the neurodegenerative diseases in the ACOS with comorbidities in our study are in accordance with these previous findings [29].

Neurodegenerative diseases [30] and ACOS [31] were chronic inflammatory disease. The exact mechanisms between ACOS and neurodegenerative diseases are not clear, however it may partially be explained by coexistence of the systemic inflammation in both ACOS [32] and neurodegenertative diseases [33]. Several studies have reported an association between an increased atherosclerosis-related [34] inflammation markers [(e.g. TNF-α [33] and an increased risk of neurodegenerative diseases [32], including stroke [34], dementia and PD [35]. The level of TNF-α increased in the ACOS cohort [36] and its value is higher than the control groups [32]. This finding may support the inflammation markers associated with the neurodegenerative diseases. Meanwhile, the IFNγ may aggravate the astherosclerosis [24] and associated with the stroke [37] even in the lower level of the ACOS cohort compared with the control group [36]. Moreover, the IFNγ with the synergistic contribution of TNF-α in PD [38]. These speculations may explain the risks of the stroke with young age (≧40 years < 65 years) and stroke, PD, dementia with elderly (> 65 years) in ACOS cohort were higher than the non-ACOS cohort in our study. However, no significant differences of the risk of the PD and dementia in the ACOS cohort with the aged > 40 years < 65 years. The role of the cytokine in the different subgroup (≧40 years < 65 years, > 65 years) of the neurodegenerative diseases among the ACOS cohort warrant further study.

Meanwhile, as previous mention [39–41], the oxidative stress of the chronic obstructive airway disease associated neurodegenerative diseases [39]. Similarly, the severe form of the ACOS with high frequency of AE and the hospitalization [16]; thus, the intermittent hypoxemia [42] and oxidative stress aggravate the exists the atherosclerosis [34] of system artery (e.g. carotid artery). Furthermore, the hypercapnia may have impact on the dementia and ventilator impairment in the PD [43]. These vehicle cycle may enhance the hypoxemia/hypercapnia [44]; thus, the incident of neurodegenerative diseases increased. The aging [45] and atherosclerosis-related diseases of ACOS cohort are associated with the system inflammation [31] also. Considering these speculations [30, 35, 39]; aging, hypoxemia [16, 42] and system inflammation [14, 31] are predisposing factors [33] for neurodegenerative diseases (e.g. stroke) even without the comorbidities.

The ICSs may be administered for treating the mild to moderate type of ACOS [13, 46]. The ICSs [47] may improve poor lung function [28]; thus, attenuating the hypoxemia level [48]. In a korea [49] and Japan [50] study shows ACOS shows the possibility of recovering their lung function under ICSs in line with this finding. Meanwhile, ACOS seems to be more responsive to bronchodilators [51] and ICSs [52] compared with the pure COPD cohort. In Feng et al, study [52], they found that ICSs (e.g. 2 mg × 3/day × 180 days = 1.08 gram) may attenuate the inflammation, hypoxemia, and hypercarpenia. These findings support the ICSs > 0.13gram in ACOS cohort may attenuate the airway and system inflammation. Thus, the incidences of the neurodegenerative diseases were less in the ACOS cohort comparison of the non-ACOS cohort. Similarly, Liu et al, report the ICSs (e.g. > 0.0395 gram) have the impact on attenuation of the chronic system inflammation in the COPD support this finding [53].

Impaired lung function is an independent predictor of cognitive diseases, such as dementia; however, evidence of its association with cognitive decline is mixed [54]. The OSs [16, 55] are used for treating the relatively severe type [55] of ACOS [13, 20]. This type involves poor lung function and more severe hypoxemia [27]. However, the eosinophilic inflammation induced by ACOS responded well to OSs. Therefore, adequate dose of the OSs use may reduce hypoxemia [56], and patients receiving steroids may have a lower risk [16] of atherosclerosis of the blood vessels [16] or amyloidosis of the brain [4]. The effect of the pulse predinsolone dose of 5 days (40 mg /days × 5days = 200 mg) [57] to 14 days (560 mg) attenuate the AE-COPD in previous study support this speculation [58]. In addition, the pulse therapy of the steroid such as dexamethasone [59] or corticosteroid [60] in previous studies may play a role in the protective effect of the dementia and PD support with these results. The use of OSs with > 0.08 gram (80 mg) were associated with the less risk of neurodegenerative diseases in our study agree these findings.

The use of OSs with < 0.07 gram [57] were associated with the higher risk of neurodegenerative diseases. Meanwhile, the ICSs < 0.12 gram were associated with the higher risk of PD. This imply that the small dose of steroid without effect on the risk of the neurodegenerative diseases. Moreover, the ACOS cohort without the ICSs /OSs were with the higher risk of the neurodegenerative diseases. In previous study, the optimal higher level dose of the dexamethasone 0.001 gram/kg (equlvalent to 0.0067 gram prednisolone/kg, e.g., 0.0067 gram × 50 kg = 0.335 gram > 0.08 gram) diminished a dopamine content depletion in striatum by about 20%, whereas the lower doses of 0.0001gram/kg (0.0335 gram was < 0.08 gram) ineffective [53, 59], this report is in line with our result. Meanwhile, the lower level of the ICSs (e.g. < 0.0395 grams) didn’t attenuate the chronic system inflammation among the COPD in observer study [53]. Similarly, the higher cumulative dose of ICSs (1.2 mg/day x 180day = 0.21 gram > 0.13gram) have a protective effect of the system inflammation (IFNγ, TNF-α), [61] in COPD-related disease such as lung cancer [62], this protective effect was not found in the lower dose (< 0.21 gram). Therefore, protective effect of ACOS combined with the higher levels of ICSs/OSs, and the risky effects of ACOS combined with the lower levels ICSs/OSs for system inflammation diseases-neurodegenerative diseases such as in current study. Similarly, in Yamauchi et al study, it appears that greater amounts of administered OSs/ICSs [63] are related to better prognosis in the ACOS cohort than in the COPD-alone cohort [64]. These speculations in need of randomized, double-blind, placebo-controlled pilot trial for confirmation.

Regarding the daily dose of the of a fluticasone equivalent ICSs [65], we found the dose [250 μg/d-500 μg/d (0.25 mg/d–0.5 mg/d) and > 500 μg/d (0.5 mg/d)] without association with the risk of the neurodegenerative diseases. Meanwhile, we also found the risk of stroke and dementia were the lower in patients with low-dose use less than 250 μg/d (0.25 mg/d) of a fluticasone equivalent ICSs [66]. As mention before, ICSs > 0.13 gram in ACOS cohort may attenuate the airway and system inflammation. Therefore, if we use the low dose of the ICSs up to 521 days, the cumulative dose of ICSs is > 130 mg [(0.13 gram)/0.25 mg/d = 520 days]. Thus, the ACOS cohort may have the less risk of the stroke and dementia. In versa, if we use the middle or high daily dose ICSs for the severe ACOS cohort with relatively short period (e.g., 240 days); thus, we shift the ICSs to OSs (e.g., from the day 241). Under this policy, the cumulative dose of ICSs with 130 mg (0.13 gram) is inaviable (e.g.,0.5 mg/d × 240 d =120 mg < 0.13 gram). Thus, the high daily dose (high up to 500 μg/d) with 240 days without significant impact on the risk of the neurodegenerative diseases. This imply that impact for the ‘dose–response’ is more efficient of the ‘cumulative dose’ than of the ‘daily dose’ between the ICSs and neurodegenerative diseases among the ACOS cohort comparison of the non-ACOS cohort. These speculations warrant randomized control trial for confirmation.

This finding of our study may alert the cliclican to early detect the ACOS [67] among the COPD or asthma groups, and we may try the adequate dose of the ICSs/OSs (e.g. minimal effect dose 0.13 grams /0.08 grams) within optimal duration (e.g., high dose in 5–7 days for OSs [57] in exacerbation of ACOS, lower dose of ICSs in daily care of ACOS for 520 days or more [52]) for avoiding the risk of the neurodegenerative diseases in the late course of the ACOS cohort. In practice, this finding imply the low dose of [0.25 mg/d of a fluticasone equivalent ICSs] combined with long course (e.g., 520 days) of ICSs may have the less incidence of the stroke and dementia. However, the steroid [68] may have the less effect on the attenuation of the hypoxemia in the steroid-resistant in asthma [69] or ACOS [20, 68]. Nevertheless, the large dose of steroid is a predisposing factor of the adverse reaction such as hypertension. Take into these findings together, the adequate dosage of steroid and the optimal duration of the steroid use warrant needs urgent resolution in future [70].

### Strengths

In ACOS cohort of our study, the mean age is 65.6 years [25], 26.5% with ICSs and 77.4% with OSs use imply that the largely portion of these patients fit the criteria of the steroid use (post-bronchodilator increase in the forced expiratory volume in one second (FEV1) >12% and > 200–400 ml from baseline) [71] which is a major component of the ACOS [68]. These characterization in line with the recent study of the ACOS in general population [72]. According to our review of relevant literature published in the English language, this study is the first to focus on the bridging the neurodegenerative diseases with the ACOS. Neurodegenerative diseases and ACOS are diagnosed using a well-established system. In this study, we analyzed patients with smoking- and alcohol-related lifestyle diseases; we enrolled patients with atopic-related diseases, which are related to ACOS also. Moreover, we considered the use of drugs, such as OSs, ICSs. The analysis were based on the combination of ACOS and ICSs/OSs use may avoid diagnosis-related bias. This novel finding warrant further investigation.

### Limitations

ACOS diagnosis is challenging for physicians. However, our study cohort was derived from the COPD cohort; more than half of the COPD diagnosis was based on the pulmonary function test (PFT) conducted in Taiwan [14]. COPD diagnosis is the result of a holistic decision-making strategy that takes into account medical history, risk factors, physical examination, spirometry, radiographic examinations and long-term response to inhaled bronchodilators and/or corticosteroids [73]. Either diagnosed COPD with PFT or COPD without PFT having related respiratory symptoms (breathlessness, ongoing cough, cough with sputum, wheezing, and chest tightness) of 3 months duration that also fulfill an epidemiological case definition of COPD [74]. In addition, the largely portion of the patients of ACOS cohort received the ICSs/OCSs. The [ICD-cohort + COPD medicine] may avoid the bias of diagnosis of COPD [75]. However, the justifications such as clinical symptoms, office spirometry, the patients received the true dosage of the medicine and specialist’s correspondence may be the limitations in this study [76]. Meanwhile, we replace the current smoking status or smoking index which were unavailable in the NHIRD by the smoking-related disease (e.g. comorbidities). In addition, the classify the dementia and PD may challenge the clinical physician. The pathology of the neurodegenerative diseases were unavailable in the NHIRD also. These points may be a cofounder for misclassification of dementia and PD. Furthermore, antihypertensive and oral hypoglycemic drugs may affect the neurodegenerative diseases. We did not analyze the effects of these drugs in this study. Thus, the role of OSs, ICSs in the neurodegenerative diseases warrant randomized controlled trials in other country for confirmation.

Campbell and Stanley (1963) delineated two types of validity: internal validity as a characteristic of the experimental treatment effect [77], and external validity that provides the basis for generalizability to other populations, settings, and times. First, issues related to the external validity of trials [78, 79] involving patients with multiple chronic conditions (stroke, PD, and dementia) of this study addressed to other area including: 1) patient factors: lack of clarity around participant inclusion and definitions if the setting didn’t have the strict NHIRD, heterogeneity and age of participants with multiple chronic conditions. 2) system factors: chronic disease interventions designed around single conditions, Secondly; patient populations (age > 20, < 40 years vs. age > 40 years) and clinicians (chest physician vs. neurologist) will be quite different in different settings. Thirdly; regarding the drug treatment, 1) Pretest-Treatment-Pretesting may sensitize the experimental subjects to the experimental factor so the results obtained can be generalized only to other pretested groups (e.g. the patients with expericine of ICSs/OSs). 2) Multiple-Treatment Interaction/Interference: subjects received more than one treatment (the patients with ICSs/OSs) [80]. 3) Selection-Treatment Interaction: the effect of the treatment of patients aged > 40 years cant not be addressed to patients < 40 years. 4) the education level of the patients living in the country is different from the city. 5) Specificity of variables: the different measuring methods of evaluation the progress of the ACOS change over time [81]. 6) Experimenter Effects: the patients received the management under the different chest physician with different dose of ICSs/OSs. 7) Reactive Arrangements: subjects may react according to their knowledge of the experiment (e.g. the skill of inhaler use among the patients enrolled into study) [78].

## MATERIALS AND METHODS

### Data source

Data were retrieved from Taiwan’s Longitudinal Health Insurance Database 2000 (LHID2000), an insurance claim database of Taiwan’s National Health Insurance (NHI) program launched in 1995. The NHI covers 99% of the Taiwanese population (approximately 24 million residents) and releases the NHI Research Database (NHIRD). The LHID2000 randomly selected 1,000,000 insurants enrolled in the NHI program from 1996 to 2000 and traced their medical utilization. Details of the LHID2000 and NHI program have been described elsewhere [82].

### Data availability statement

The dataset used in this study is held by the Taiwan Ministry of Health and Welfare (MOHW). The MOHW must approve our application to access this data. Any researcher interested in accessing this dataset can submit an application form to the MOHW requesting access. Please contact the staff of MOHW (Email: stcarolwu@mohw.gov.tw) for further assistance. Taiwan MOHW Address: No.488, Sec. 6, Zhongxiao E. Rd., Nangang Dist., Taipei City 115, Taiwan (R.O.C.). Phone: +886-2-8590-6848. All relevant data are within the paper.

### Ethics statement

The NHIRD encrypts patient personal information to protect privacy and provides researchers with anonymous identification numbers associated with relevant claims information, including sex, date of birth, medical services received, and prescriptions. Therefore, patient consent is not required to access the NHIRD. This study was approved to fulfill the condition for exemption by the Institutional Review Board (IRB) of China Medical University Hospital (CMUH104-REC2-115-CR2). The IRB also specifically waived the consent requirement.

### Study design and participants

We recruited patients with new diagnosis of the COPD [International Classification of Diseases, Ninth Revision, Clinical Modification (ICD-9-CM) 491, 492, and 496) who were aged ≥ 40 years [13] and had concurrent physician-diagnosed new asthma (ICD-9-CM 493) [72] from January 1, 2000 to December 31, 2010 in the ACOS cohort. These patients diagnosed as having asthma in ≥ 3 outpatient and/or hospitalizations visits and COPD in ≥ 3 outpatient and/or hospitalizations visits. The index date was defined as the date of new asthma diagnosis. We excluded patients with missing age, sex data, and those with stroke (ICD-9-CM 430–438), PD (ICD-9-CM 332), and dementia (ICD-9-CM 290, 294.1, and 331.0) had been diagnosed prior to the diagnosis of COPD, or the patients had cases of asthma before the index date. For each patient with ACOS, two non-ACOS comparison patients were frequency-matched for sex, age (5-year intervals), and ACOS diagnosis year. The index date for patients without ACOS were the date of randomly assigned month and day with the same index year of the patients with ACOS. These patients follow up to diagnosis of stroke, PD and dementia; or the December 31, 2011. The strict policy of the NHIRD data based on the below: 1) nearly 58.4% of COPD received the PFT in Taiwan. 2) under the policy of releasing long-term prescription and escalating public medical knowledge to prevent over-use of National Insurance Resource, we need follow up the PFT [14]. 3) The policy of releasing long-term prescription with ICSs in COPD and grading of severity of airflow limitation in COPD based on post-bronchodilator forced expiratory volume in 1 second (FEV1) were frequently used in Taiwan [FEV1/forced vital capacity (FVC) < 0.7] based on the post-bronchodilator increase in FEV1 >12% and > 200–400 ml from baseline [83]. 4) The justification of the drug use of new diagnosis of COPD [84] or Integrated care for geriatric frailty [85] in ACOS cohort based on the PFT is popular in Taiwan. For example, 61% hypertension and 39.6% of CAD among the ACOS cohort, we need PFT for evaluation of the beta-blocker use [86] which may be used carefully in the disease containing of the asthma component such as ACOS.

### Outcome, comorbidities

Both ACOS and non-ACOS cohorts were followed until one of the following events occurred: the diagnosis of stroke, PD, or dementia; patient data being censored because of withdrawal from the NHI program; or the end of 2011. The baseline comorbidity history for each participant was determined before index date. The smoking and atopic diseases were associated with the ACOS [49]. Therefore, the preexisting comorbidities-related [87] with smoking [e.g. diabetes (ICD-9-CM 250), hypertension (ICD-9-CM 401–405), hyperlipidemia (ICD-9-CM 272), coronary artery disease (ICD-9-CM 410–414), alcohol-related illness (ICD-9-CM 291, 303, 305, 571.0, 571.1, 571.2, 571.3, 790.3, A215, and V11.3), sleep disorder (ICD-9-CM 307.4 and 780.5), anxiety (ICD-9-CM 300.00), cancer (ICD-9-CM 140–208), tuberculosis (ICD-9-CM 010–018), pneumonia (ICD-9-CM 481–486)] and atopic-related diseases were analyzed.

### Exposure assessment

The use of drugs, namely ICSs/OSs [56, 88], was analyzed. The ICSs that were analyzed in this study included fluticasone propionate and budesonide. Information regarding exposure to ICSs was extracted from the prescription database. Users of ICSs/OSs were defined as those who received at least one prescription for ICSs/OSs between the ACOS diagnosis date and index date. The date of prescription, and number of days supplied were identified. The use of ICSs/OSs were approved in Taiwan in January, 2001 and was placed on the listing of NHI drugs for reimbursement in February, 2002. The (ICSs, OSs) users were defined as patients who received prescription for ≧ (30, 5) days, and those who did not receive ICSs/ OSs prescription were classified as nonusers of ICSs/OSs. Any ICSs/OSs, prescribed after index date and before the endpoints for the subjects was considered as exposure. We calculated recorded average dose of ICSs/OSs therapy per year by dividing the total prescribed dose by the follow-up period. The average dose on median use is partitioned in to 3 segments by tertile of ICSs/ OSs. Moreover, we categorized the patients with ICSs used into four groups on the basis of daily equivalent fluticasone doses: no fluticasone use, low dose (< 250 μg/d), medium dose (250–500 μg/d), and high dose (> 500 μg/d).

### Statistical analysis

The distribution of characteristics between the ACOS and non-ACOS cohorts showed means and corresponding standard deviations (SDs) for age and determined the number and percentage for sex, age group, comorbidities, and drugs used. The cumulative incidence of stroke, PD, or dementia was assessed using the Kaplan–Meier method, and the curve difference was determined using the log rank test. The incidence density rates of stroke, PD, or dementia stratified by sex, age, comorbidities, and drugs used were calculated in both cohorts. Univariable and multivariable Cox proportional hazards regression models were used to estimate the hazard ratios (HRs) and 95% confidence intervals (CIs) for stroke, PD, or dementia in the ACOS cohort compared with the non-ACOS cohort. Furthermore, the multivariable Cox proportional hazards regression model was used to estimate the risk of stroke, PD, or dementia on the basis of the drugs used for treatment. SAS Version 9.4 (SAS Institute, Cary, NC, USA) was used for data management and statistical analyses. A two-sided *P* value of < .05 was considered significant.

## CONCLUSIONS

The ACOS cohort had a higher risk of the neurodegenerative diseases. The lower dose of the inhaler corticosteroids with cumulative dose ≧0.13 gram have the less risk of stroke and dementia.

## Abbreviations

ACOS: asthma–chronic obstructive pulmonary disease syndrome
PD: Parkinson's disease
aHR: adjusted hazard ratio
CI: confidence interval
OS: oral steroid
IC: inhaled corticosteroid
CAD: coronary artery disease
TB: tuberculosis
COPD: chronic obstructive pulmonary disease
NHIRD: National Health Insurance Research Database
LHID 2000: Longitudinal Health Insurance Database 2000
ICD-9- CM: International Classification of Diseases, Ninth Revision, Clinical Modification
